# The ER stress regulator Bip mediates cadmium-induced autophagy and neuronal senescence

**DOI:** 10.1038/srep38091

**Published:** 2016-12-01

**Authors:** Tao Wang, Yan Yuan, Hui Zou, Jinlong Yang, Shiwen Zhao, Yonggang Ma, Yi Wang, Jianchun Bian, Xuezhong Liu, Jianhong Gu, Zongping Liu, Jiaqiao Zhu

**Affiliations:** 1 College of Veterinary Medicine, Yangzhou University, Yangzhou, 225009, PR China; 2 Jiangsu Co-innovation Center for Prevention and Control of Important Animal Infectious Diseases and Zoonoses, Yangzhou, 225009, PR China; 3 Jiangsu Key Laboratory of Zoonosis, China

## Abstract

Autophagy is protective in cadmium (Cd)-induced oxidative damage. Endoplasmic reticulum (ER) stress has been shown to induce autophagy in a process requiring the unfolded protein response signalling pathways. Cd treatment significantly increased senescence in neuronal cells, which was aggravated by 3-MA or silencing of Atg5 and abolished by rapamycin. Cd increased expression of ER stress regulators Bip, chop, eIf2α, and ATF4, and activated autophagy as evidenced by upregulated LC3. Moreover, the ER stress inhibitor mithramycin inhibited the expression of ER stress protein chaperone Bip and blocked autophagic flux. Downregulating Bip significantly blocked the conversion of LC3-I to LC3-II, decreased LC3 puncta formation, and prevented the increase of senescence in PC12 cells. Interestingly, knocking down Bip regulated the expression of p-AMPK, p-AKT and p-s6k induced by Cd. BAPTA, a Bip inhibitor, decreased the expression of p-AMPK and LC3-II, but enhanced neuronal senescence. In addition, we found that siRNA for Bip enhanced GATA4 expression after 6 h Cd exposure in PC12 cells, while rapamycin treatment decreased GATA4 levels induced by 24 h Cd exposure. These results indicate that autophagy degraded GATA4 in a Bip-dependent way. Our findings suggest that autophagy regulated by Bip expression after ER stress suppressed Cd-induced neuronal senescence.

Cadmium (Cd) has been reported as a significant toxic and carcinogenic element that is widely present in the environment^1^. Cd targets several organs and tissues such as kidney^2^, blood^3^, bones^4^, testis^5^, and brain^6^. Acute Cd poisoning results in Parkinsonism^7^, and Cd intoxication has been identified as a potential factor in neurodegenerative diseases such as Parkinson’s disease (PD) and Alzheimer’s disease (AD)^8^.

It has been reported that Cd causes DNA damage in cerebral cortical neurons^9^. Some recent reports indicate that cultured neuronal cells undergo apoptosis when exposed to relatively high doses of Cd^9^,^10^. In addition, exposure to such a dose of Cd reportedly causes marked ROS accumulation and autophagy in cultured neurons^10^. However, apoptosis is not the major cause for neuron damage in the AD brains^11^, where the loss of neurons and their functional plasticity impairment by synaptic changes such as premature senescence is considered to play the key role^12^. Intriguingly, recent data show that low, clinically-relevant doses of DNA damaging drugs do not induce cellular apoptosis but instead lead to the permanent growth arrest associated with cellular senescence^13^,^14^.

Despite the fact that cellular senescence in peripheral tissues has recently been linked to a number of stress pathologies, its involvement in neurodegeneration is just beginning to be explored. ROS, DNA damage, cytokines and oncogenic activation can all aggravate cellular senescence, and this phenomenon is termed stress-induced premature senescence^15^,^16^. These findings suggest that smaller oligomeric, misfolded protein aggregates or larger fibrillar aggregates can lead to neuronal senescence^17^. In our previous studies examining Cd as a vital stress factor, Cd induced ROS in neurons^18^; these ROS can be involved in a range of events from proliferation to growth arrest or senescence^19^.

A senescent neuron is defined functionally by its inability to respond appropriately to growth factors and by its expression of senescence-associated proteins^20^. Replicative senescence/permanent cell cycle arrest was previously identified as an important mechanism controlling normal cell proliferation, and the altered expression of senescence-specific markers^21^. Moreover, recent studies have revealed a remarkable connection between inflammatory mediators and senescence. These studies demonstrate that a hallmark of physiologically senescent cells is a massively increase in the secretions of multiple proinflammatory proteins, including IL-6, IL-8 (CXCL8) and other chemokines and cytokines^22^,^23^,^24^. Therefore novel anti-inflammatory approaches need to be designed to reduce the paracrine effects of the inflammation to limit the spread of neurodegeneration, and limit the collateral damage caused by Cd.

Macroautophagy, hereafter referred to as autophagy, is defined as a lysosomal pathway that degrades and recycles intracellular organelles and proteins to maintain energy homeostasis during times of nutrient deprivation, and to remove damaged cell components^25^,^26^. Altered autophagy has been implicated in AD and many other neurodegenerative conditions^27^. In addition, monitoring of autophagic flux includes assessment of p62 degradation and the activity of autolysosomal hydrolases^28^, as well as examination of the quenching of GFP-tagged LC3 protein^29^,^30^. Autophagy is regulated by AMPK signalling^31^,^32^. The most commonly described mechanism is suppression of the mTORC1 pathway^31^,^33^,^34^. The role of AMPK in preventing aging/senescence has also been suggested in many studies^35^,^36^,^37^. However, the specific mechanism remains unclear.

The endoplasmic reticulum (ER) is a dynamic network of interconnected membrane tubules that essentially reaches every part of the cell, including dendrites and axons in neurons^38^. Accumulating data suggest that endoplasmic reticulum (ER) and autophagy are cross regulated; ER stress leads to the activation of self-protective mechanisms that include the unfolded protein response (UPR) and autophagy to avoid cell damage via 3 UPR pathways^39^,^40^,^41^. However, under conditions of severe ER stress, this mechanism may also activate cell death programs^42^,^43^. A major UPR-upregulated target protein is the 78 kDa glucose-regulated protein GRP78, an ER molecular chaperone also known as Bip, which is a key regulator of ER stress transducers^44^. During ER stress, Bip is induced to increase the folding capacity of the ER and to compensate the depletion of free Bip^45^, which may have anti-senescent effects involving multiple mechanisms^46^,^47^. Cd induces ER stress in various cell types^48^,^49^,^50^, but the factors and mechanisms underlying Cd-induced premature senescence by Bip in neuronal cells remain to be elucidated.

The transcription factor GATA4 is a regulator of embryonic development^51^, and is localised to regions of the mouse embryo involved in cardiac development. Recently, Kang *et al*. determined that GATA4 is abundant in aging brain, acting as an upstream regulator of NF-kB^52^. The evidence suggests that GATA4 plays a pivotal role in inflammation and is a key target for the suppression of inflammation and senescence^53^.

Autophagy is reported to be required for the establishment of senescence^54^,^55^,^56^ or to inhibit senescence^57^. Nevertheless, the relationship between autophagy and senescence is not yet fully understood. In this study, we aimed to investigate whether autophagy and ER stress regulator Bip are involved in Cd-induced senescence in neuronal cells.

## Materials and Methods

### Reagents

Foetal calf serum (FCS) was obtained from Hyclone Laboratories (Logan, UT, USA); SA-β-Gal Staining kits were obtained from Cell Signaling Technology (Boston, MA, USA). BCA protein assay kit (Nanjing, Jiang Su, China). Cell cycle analysis kit was purchased from BD Company (San Diego, CA, USA). PrimeScript™ RT Reagent kit and SYBR^®^ Premix Ex Taq were obtained from TaKaRa (Shanghai, China). TRIzol^®^ Reagent was purchased from Thermo Fisher Scientific (Waltham, MA, USA). P21, P16, P53, p62 and β-actin antibody, Tetramethylrhodamine (TRITC)-conjugated anti-rabbit IgG was purchased from Abcam Technology (Shanghai, China). RIPA lysis buffer, anti-LC3 polyclonal antibody, Neurobasal^®^ medium, B-27^®^ supplement, 4′,6-diamidino-2-phenylindole (DAPI) stain, Dulbecco’s modified Eagle’s medium (DMEM)-F12, propidium iodide (PI) and N-acetyl cysteine (NAC) were purchased from Sigma Chemical Co. (St Louis, MO, USA). Other antibodies were purchased from Cell Signaling Technology (Boston, MA, USA).

### Cells

The rat pheochromocytoma (PC12) cell line was purchased from the Cell Bank of Type Culture Collection of Chinese Academy of Sciences (Shanghai, China) and were cultured at 37 °C in 5% CO_2_ in antibiotic free RPMI 1640 medium supplemented with 10% heat-inactivated horse serum and 5% FCS. For isolation of primary neurons, primary cortical neurons were isolated from foetal mice at 18 days of gestation and cultured as described^58^. Fresh medium was replaced every 3 days. Primary neurons were used for experiments after 6 days of culture.

### Senescence-associated β-galactosidase staining assay

Senescence-associated-β-galactosidase (SA-β-Gal) staining was performed using an SA-β-Gal Staining Kit according to the manufacturer’s instructions. After Cd treatment, PC12 cells were washed in PBS, fixed for 3–5 min (room temperature) in 4% (v/v) formaldehyde and stained with SA-β-Gal staining solution (pH 6.0) at 37 °C (no CO_2_) for 16 h. Blue cells were counted under a phase-contrast microscope (Leica DMI 3000B, Solms, Germany).

### Real time analysis of cytotoxicity

Cell activity were monitored using xCELLigence real-time cell analysis (RTCA; Roche Applied Science, Basel, Switzerland) according to the manufacturer’s protolcol^59^. The background level was first determined by loading 100 μl/well of culture medium (1640 with 10% FBS) into a 16-well E-plate. PC12 cells were seeded at a density of 10,000 cells/well in 100 μL aliquots in quadruplicate and cultured for 14 h to make sure the cells adhere and reach their proliferative phase. The impedance was measured every 15 min. During the exponential phase, cells were treated according to the experimental design. The results were normalized to cell proliferation at the time of treatment.

### Analysis of cell cycle by flow cytometry

After treatment, PC12 cells were harvested by trypsinisation and rinsed in PBS before fixation in 70% ethanol overnight at 4 °C. After two additional rinses in ice-cold PBS, cells were then incubated with 1 mg/ml RNaseA in PBS at 37 °C for 30 min, then stained with 0.5 mg/ml PI in PBS at room temperature for 15 min to label intracellular DNA, and the cell cycle distribution within each sample was determined by flow cytometry. PI fluorescence was detected at 488 nm excitation and 630 nm emission then analysed with ModFit software.

### Western blotting

At the end of incubation, cells were washed with PBS, lysed in ice cold RIPA buffer, and sonicated. Protein content was determined using a BCA protein assay. Protein samples (20–80 μg) were subjected to SDS-PAGE followed by Western blotting as previously described^18^,^58^. All primary antibodies were diluted at 1:1000, the corresponding HRP-conjugated secondary antibodies were diluted at 1:5000. All assays were performed in triplicate.

### RNA extraction, reverse transcription and quantitative reverse transcription polymerase chain reaction (qRT-PCR)

Total RNA was extracted from cultured cerebral cortical neurons using TRIzol^®^ Reagent according to the manufacturer’s protocol. A total of 900 ng RNA was reverse transcribed into cDNA using a Prime Script™ RT Reagent kit with gDNA Eraser. The primers were designed using Primer Premier 5 (PREMIER Biosoft Int., Palo Alto, CA, USA.). Expression levels of all genes were measured using a real-time PCR system (Applied Biosystems 7500, USA) and the reactions were performed with a SYBR^®^ Premix Taq™ II kit according to the manufacturer’s instructions. Annealing temperatures were 60 °C for all primers. The analyses of relative mRNA levels of IL1α and IL6 were carried out using the ΔΔC_T_ method and values were normalized to an internal β-actin control.

### Immunofluorescence

Cells were seeded on gelatin-coated coverslips were fixed in 4% paraformaldehyde, permeabilised with 0.5% Triton, and blocked in 5% FBS. Primary antibodies (LC3, P21, Bip and GATA4 diluted at 1:100) were incubated with samples overnight at 4 °C followed by incubation with fluorescent secondary antibodies at 37 °C for 1 h. F-actin staining was performed following incubation with TRITC–phalloidin. DAPI was used to visualize the nuclei. All fluorescent samples were visualized with a confocal fluorescence microscope (Leica TCS SP8; Leica Corporation, Germany).

### Tandem EGFP-mRFP confocal microscopy

PC12 cells stably transfected with EGFP-mRFP-LC3B were seeded into glass bottom cell culture dishes with 1 × 10^5^ cells per dish. After treatment, the cells were washed with PBS and examined under a Leica confocal microscope system (Leica DMI 3000B, Solms, Germany).

### Electron microscopy

For transmission electron microscopy (TEM) observation, after being treated with 10 μM Cd in the presence or absence of autophagy inhibitor Chloroquine (CQ) for 6 h, the cells were collected and fixed in ice-cold glutaraldehyde (2.5% in 0.1 mol/L cacodylate buffer, pH 7.4) for 24 h and cells were postfixed postfixed with 1% OsO4. After dehydration with a graded series of alcohol concentrations, the samples were rinsed in propylene oxide and impregnated with epoxy resins. Frontal sections were cut, stained with 2% uranyl acetate in 50% ethanol and lead citrate, and examined using a PHILIPS CM-120 transmission electron microscope.

### RNA interference of ATG5 or Bip

Retroviral vectors containing ATG5-siRNA or Bip-siRNA and a negative control (NC) used in this study were constructed from Abm Company. The retrovirus-expressing blank served as a negative control. A monolayer of PC12 cells, when grown to about 70% confluence, were infected with the above retrovirus -containing supernatant in the presence of 8 mg/ml polybrene for 24 h, and then exposed to 2 mg/ml puromycin. Cells were used for Cd treatment 5 days after infection.

### Statistical analysis

Results are presented as the mean ± standard deviation (SD). Statistical data comparisons among groups were performed using a non-parametric, one way analysis of variance (ANOVA) with p < 0.05 considered statistically significant. Each experiment was performed at least in triplicate.

## Results

### Senescence induced by Cd in neuronal cells

**Figure 1:**
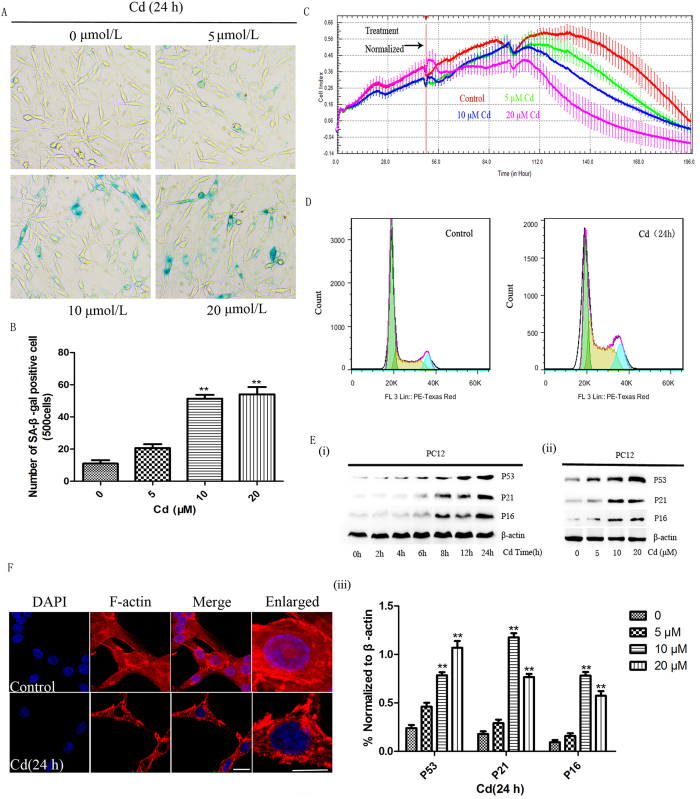
Senescence is induced by Cd in PC12 cells. (**A**) PC12 cells were treated with 0, 5, 10, or 20 μM Cd for 24 h and then subjected to an SA-β-Gal staining assay. (**B**) The number of SA-β-Gal positive cells in each group from Fig. 1A was counted and listed. (**C**) The effect of Cd on cell index (CI). Data were normalized to the effect of Cd on PC12 cells at the time of 0, 5, 10, or 20 μM Cd treatment. All curves were plotted as an average of quadruplicate treatments. Error bars indicate SD (n = 3). (**D**) PC12 cells were cultured in the presence or absence of 10 μM Cd for 24 h. The cells were harvested and stained with PI solution, and DNA content, an indication of the stage of the cell cycle, was determined by flow cytometry and analysed using Flowjo Software. (**E**) PC-12 cells were treated with 10 μM Cd for different times (i) or with 0–20 μM Cd for 24 h (ii). Cell lysates were analysed by western blot assay with a specific antibody to P53, P21 and P16. β-actin expression was used as a loading control. Blots for the target proteins were semi-quantified using Image Lab (iii). (**F**) PC12 cells were treated with or without 10 μM Cd for 24 h and were stained to visualize F-actin (**p < 0.01 compared to the control group, Scale bars: 10 μm).

To determine if Cd induces neuronal senescence, PC12 cells were subjected to a senescence associated β-gal (SA-β-Gal) assay after exposure to Cd for 24 h (Fig. 1A,B). Cd treatment significantly increased the number of positive SA-β-GAL staining cells in a dose-dependent manner. Moreover, Cd exposure also induced cytotoxicity in PC12 cells (Fig. 1C). When the concentration of Cd increased the growth slope increased less rapidly in comparison to control cells. After PC12 cells were treated with 10 μM Cd for 24 h, the percentage of S-phase cells significantly decreased while the percentages of both G0/G1- and G2/M-phase cells were increased (Fig. 1D). PC12 cells were then exposed to a sub-apoptotic dose of Cd (10 μM) for 0–24 h. Western blot analysis showed that the expression of senescence marker proteins such as P53, P21, and P16 were elevated in a time-dependent manner (Fig. 1E). PC12 cells were then exposed to increasing concentrations of Cd for 24 h (Fig. 1E). P21 and P16 protein expression peaked with 10 μM and decreased with 20 μM Cd exposure, while the expression of P53 increased in a concentration dependent manner. Another senescence marker was analysed in Cd-exposed neuronal cells. It is well known that F-actin assembly can be observed in senescent cells, so we assessed changes in F-actin organization after Cd exposure. We found that Cd treatment resulted in altered stress fibre distribution in PC12 cells (Fig. 1F).

**Figure 2:**
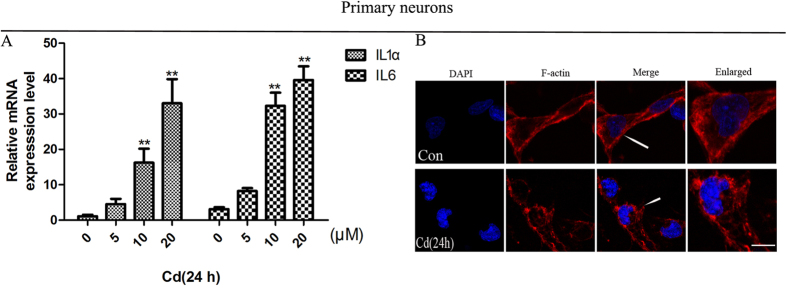
Cd induces a senescent-like phenotype in primary neurons. (**A**) Effect of 0, 5, 10, or 20 μM Cd on IL1α and IL6 mRNA expression in cortical neurons. (**B**) Primary cortical neurons were treated with or without 10 μM Cd for 24 h and then stained with TRITC–phalloidin to visualize F-actin (**p < 0.01 compared to the control group, scale bars: 10 μm).

These results support our hypothesis that the onset of neuronal senescence may be an important mechanism underlying Cd-induced neurotoxic effects. Cd induces a senescence-like phenotype in primary neurons, as shown in (Fig. 2A), as treatment with Cd increased mRNA expression of IL1α and IL6 in a concentration-dependent manner. In addition, Cd treatment altered stress F-actin distribution in primary neurons (Fig. 2B), similar to its effects on PC12 cells.

### ER stress induced by Cd triggers autophagy in neuronal cells

**Figure 3:**
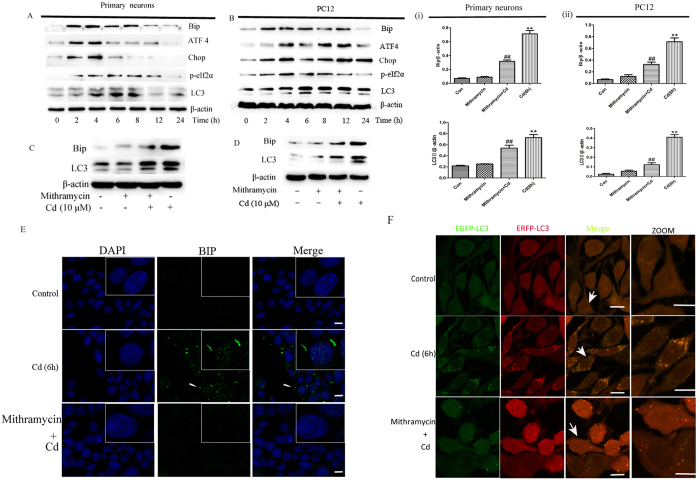
Autophagy is triggered by ER stress in Cd-treated neuronal cells. (**A**) Primary neurons or (**B**) PC12 cells were treated with 10 μM Cd for 0-24 h. Representative blots show expression of Bip, ATF4, Chop, p-eIf2α and LC3. β-actin was used as a loading control. (**C**) Primary neurons or (**D**) PC12 cells were treated with 10 μM Cd in the presence or absence of 100 nM mithramycin for 6 h. Whole cell lysates were analysed by western blotting for the expression of Bip and LC3. β-actin was used as a loading control. Blots for LC3 and Bip in primary neurons (i) or PC12 cells (ii) were semi-quantified using Image Lab. After PC12 cells were cultured in 10 μM Cd with or without mithramycin for 6 h, expression of (**E**) Cells were treated without or with to Cd (10 μM) and mithramycin (100 nM). Bip expression was analysed by immunofluorescence. (**F**) Cells were transfected with a tandem reporter construct (tfLC3) and were exposed to Cd (10 μM) and mithramycin (100 nM) as indicated in (**C**). The colocalization of EGFP and mRFP-LC3 puncta was examined by confocal microscopy (**p < 0.01 vs control group, ^##^*P* < 0.01 vs Cd group, scale bars: 10 μm).

We previously reported that Cd induced autophagy in primary neurons^58^, and investigated if ER stress is associated with Cd-induced autophagy. Treatment of primary neurons or PC12 cells with 10 μM Cd for 0–24 h resulted in changes in the expression of p-eIF2α, Bip, chop, ATF4 and LC3 (Fig. 3A,B), indicating that neuronal cells exposed to Cd undergo both ER stress and autophagy. To determine if ER stress induced by Cd leads to formation of autophagic vesicles, we exposed primary neurons and PC12 cells to mithramycin, an ER stress inhibitor, before Cd exposure. Mithramycin treatment blocked the increase in Bip and abrogated the significant upregulation of LC3 induced by Cd at 6 h (Fig. 3C,D). The inhibition of ER stress was further confirmed by Bip immunofluorescent staining, which confirmed that increased expression of Bip induced by Cd was blocked by mithramycin (Fig. 3E).

To further examine whether the effects of ER stress induced by Cd are associated with the regulation of autophagic flux, cells were transfected with a tandem reporter construct (tfLC3) and treated with Cd followed by assessment of EGFP-LC3 and mRFP-LC3 colocalisation. Exposure to Cd caused pronounced formation of LC3 puncta that displayed both green and red fluorescence producing a yellow overlay, and this colocalisation was inhibited by mithramycin (Fig. 3F). These results support our hypothesis that ER stress is involved in the regulation of Cd-mediated autophagy in neuronal cells.

### Autophagy inhibition increases senescence in neuronal cells

**Figure 4:**
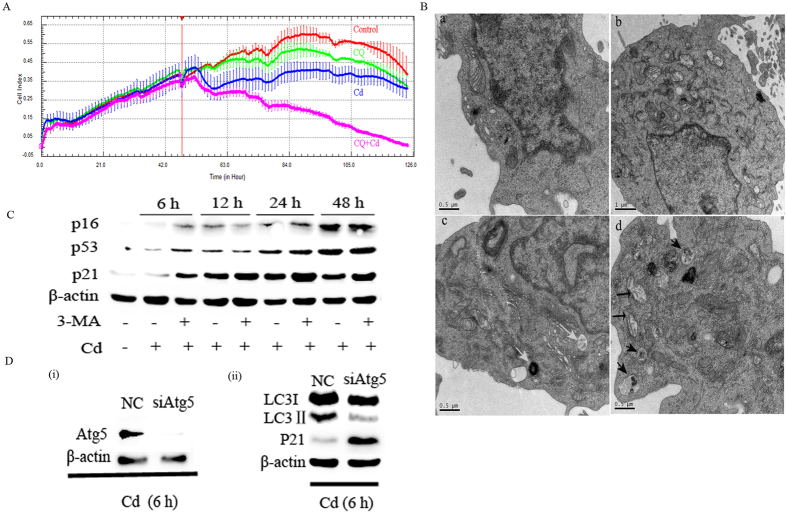
Autophagy inhibition increases senescence in neuronal cells. (**A**) Cell index for cells treated with 10 μM Cd and 5 μM CQ as indicated. Results were normalized to the time of treatment. (**B**) PC12 cells were cultured without or with Cd in the presence or absence of CQ (a: control, b: CQ, c: Cd, d: CQ + Cd) were subjected to TEM analysis. Degrading autophagic vacuoles are highlighted by white arrows in c. Damaged mitochondria (black arrows) and intermediate autophagic vacuoles (black arrowheads) are highlighted in d. (**C**) PC12 cells were treated with or without 3-MA for 6, 12, 24 or 48 h in the presence of Cd, and the relative levels of P16, P21 and P53 were determined by Western blotting. (**D**) PC12 cells were transfected with 40 nM siNC or siRNA directed against Atg5 (siAtg5) and were exposed to Cd for 6 h. Cell lysates were analysed for Atg5 and β-actin (i) or LC3, P21 and β-actin expression by western blot using antibodies sequentially (ii). The experiments were repeated two times.

To examine whether autophagy plays a role in senescence induced by Cd, an autophagy inhibitor, CQ, was used. Culturing with 10 μM Cd in the presence of 5 μM CQ decreased the CI value significantly compared with the Cd group (Fig. 4A). In addition, CQ increased intermediate autophagic vacuoles in mitochondria in PC12 cells exposed to Cd (Fig. 4B). We also assessed the expression of p16, p53 and p21 in PC12 cells cultured with Cd for 0–24 h in the presence or absence of 3-MA, a specific autophagy inhibitor (Fig. 4C). The expression of these proteins increased in a time-dependent manner, and was further increased by 3-MA. In addition, we treated siAtg5 and negative control-transfected cells with Cd (Fig. 4D). siAtg5 decreased Atg5 expression after Cd exposure. Cd treatment in the presence of Atg5 deficiency inhibited LC3 conversion but increased expression of P21 when compared to the cells treated with Cd. Together, these finding suggest that neuronal senescence was increased by autophagy inhibition.

### Rapamycin suppressed Cd-induced premature senescence in PC12 cells

**Figure 5:**
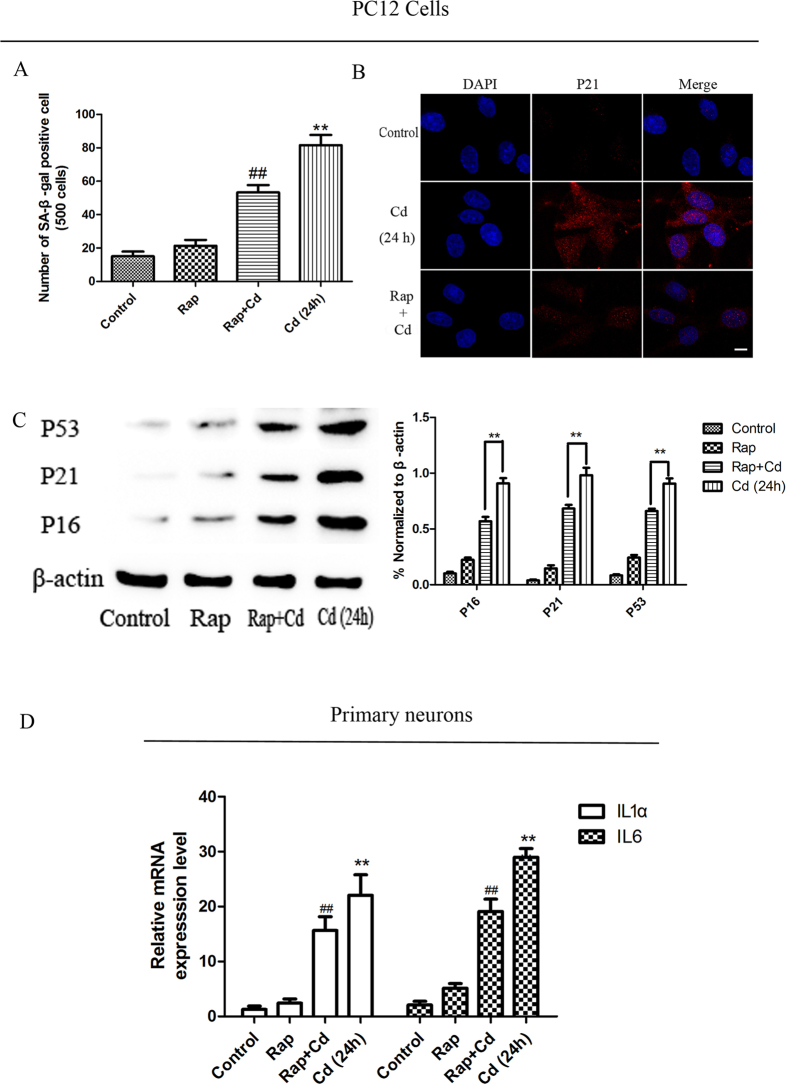
Rapamycin suppressed Cd-induced premature senescence in PC12 cells. (**A**) PC-12 cells were pretreated with 100 nM rapamycin for 24 h, followed by treatment with Cd for another 24 h, and were assayed for SA-β-Gal staining. (**B**) Effect of Cd on P21 expression in the presence or absence of rapamycin was determined by indirect immunofluorescence. (**C**) Western blot analysis for P53, P21 and P16 protein expression. The blots were probed for β-actin as a loading control. (**D**) Primary neurons were treated as indicated in (**A**), and IL1α and IL6 mRNA expression were analysed by RT-PCR (**p < 0.01 versus control group; ^##^p < 0.01, versus from Cd group).

Pretreatment with rapamycin significantly reduced the number of SA-β-Gal positive cells following Cd treatment for 24 h (Fig. 5A). Rapamycin pretreatment reduced the Cd-induce increase in p21 in PC12 cells (Fig. 5B,C). In addition, rapamycin also attenuated Cd-induced elevations in p53 and p16 expression in PC12 cells (Fig. 5C). As interleukin-mediated inflammation has been implicated in neural senescence^17^, we measured the mRNA expression of IL1α and IL6 in primary neurons exposed to Cd for 24 h with or without rapamycin. Expression of both IL1α and IL6 induced by Cd was suppressed by rapamycin (Fig. 5D). Our findings suggest that autophagy protected neuronal cells against Cd-triggered premature senescence.

### Knocking down Bip inhibits autophagy activation while aggravating senescence induced by Cd in neuronal cells

**Figure 6:**
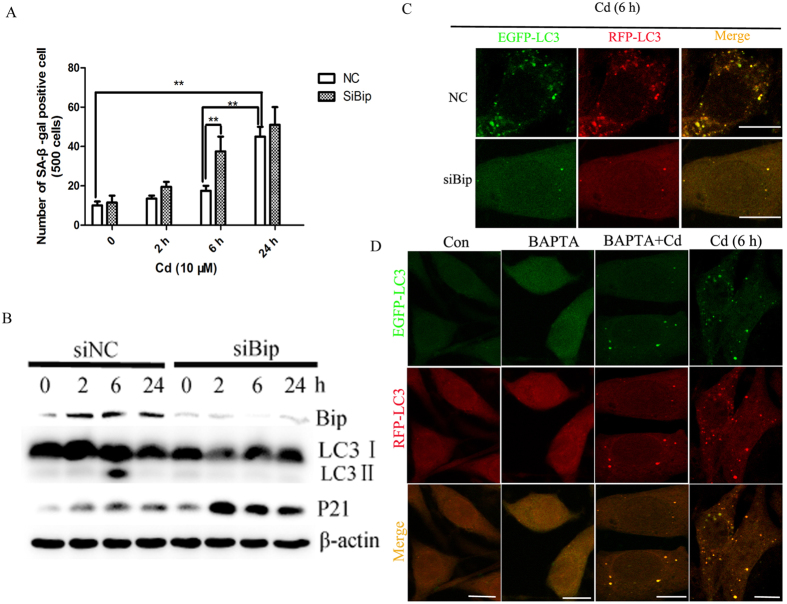
Bip knockdown blocks autophagosome formation and promotes senescence. (**A**) Effect of siBip on the number of SA-β-Gal positive cells cultured with 10 μM Cd for 6 h. (**B**) PC12 cells were transiently transfected with GFP-RFP-LC3 which were then exposed to Cd (10 μM) in combination with siNC or siBip (**B**) or 100 nM BAPTA (**C**) for 6 h. (**D**) PC12 cells were transfected with siNC or siBip and subjected to Cd treatment for 0, 2, 6, or 24 h. Western blot analysis of Bip, LC3, P21 and β-actin (as loading control) protein levels in cells.

To investigate the role of Bip in autophagy against senescence, we transfected PC12 cells with Bip siRNA or negative control siRNA (NC). Cd-induced senescence and autophagy pathways were then examined in this Bip knockdown model. Depletion of Bip by siRNA increased the number of SA-β-Gal positive cells induced by Cd at 6 h (Fig. 6A). Western blot analysis of PC12 cells transfected with Bip siRNA identified a marked decrease in Bip expression, downregulated LC3II/LC3I conversion and enhanced expression of P21 compared with NC in the presence of Cd (Fig. 6B). The effect of Bip downregulation on autophagy was evaluated by GPF-RFP-LC3 puncta formation in PC12 cells using confocal microscopy. Both siBip and BAPTA decreased Cd-induced LC3 puncta formation (Fig. 6C,D). These data further confirm that autophagy inhibits Cd-induced senescence and is dependent on activation of Bip in PC12 cells.

### Activation of AMPK-mTOR is associated with Bip mediated autophagy

**Figure 7:**
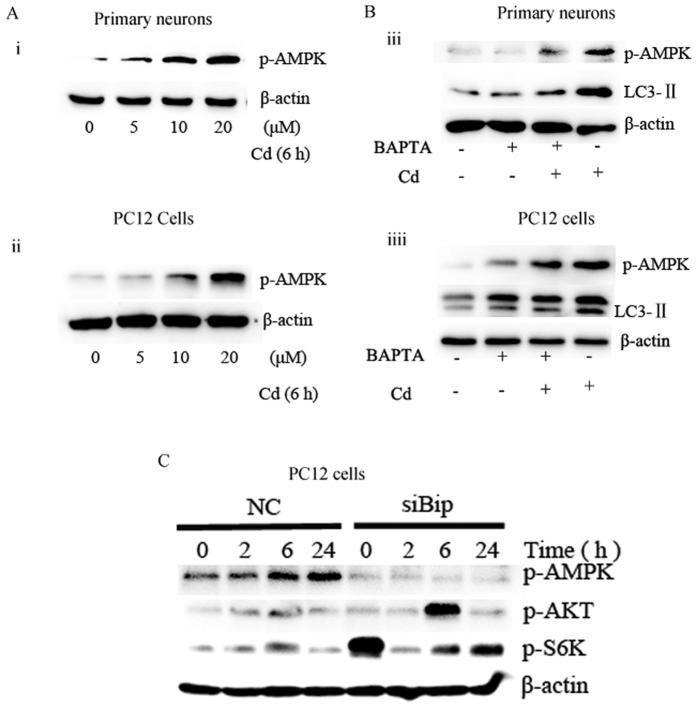
Activation of AMPK-mTOR is associated with Bip-mediated autophagy. (**A**) Effect of Cd on p-AMPK (Ser217) in (i) primary neurons, (ii) PC12 cells. (**B**) Representative blots for p-AMPK and LC3B in (iii) primary neurons, (iiii) PC12 cells treated with 10 μM Cd. (**C**) Expression of p-AMPK, p-AKT and p-S6K after siRNA silencing of Bip in PC12 cells for 0, 2, 6, or 24 hours. β-actin was used as a loading control.

We wanted to know whether the activation of AMPK-mTOR occurred in our model. Cd phosphorylated AMPK in primary neurons and PC12 cells (Fig. 7A). To confirm the role of Bip in AMPK activation, we exposed the cells to BAPTA before treating with Cd. Pretreatment with BAPTA inhibited Cd-induced conversion of LC3-I to LC3-II, and blocking Bip activation suppressed the activation of AMPK (Fig. 7B). Considering the already known relationship between mTOR and autophagy^60^,^61^, we detected the expression of P-AMPK to confirm if Bip induces autophagy through AMPK-mTOR by using specific Bip siRNA (Fig. 7C). Importantly, depletion of Bip by BipsiRNA apparently abolished the activation of AMPK, but enhanced the expression of P-AKT and P-P70s6k, which indicated that the activation of AMPK-mTOR is associated with Bip mediated autophagy.

### Autophagy suppresses GATA4 in Bip-dependent way in Cd cultured neuronal cells

**Figure 8:**
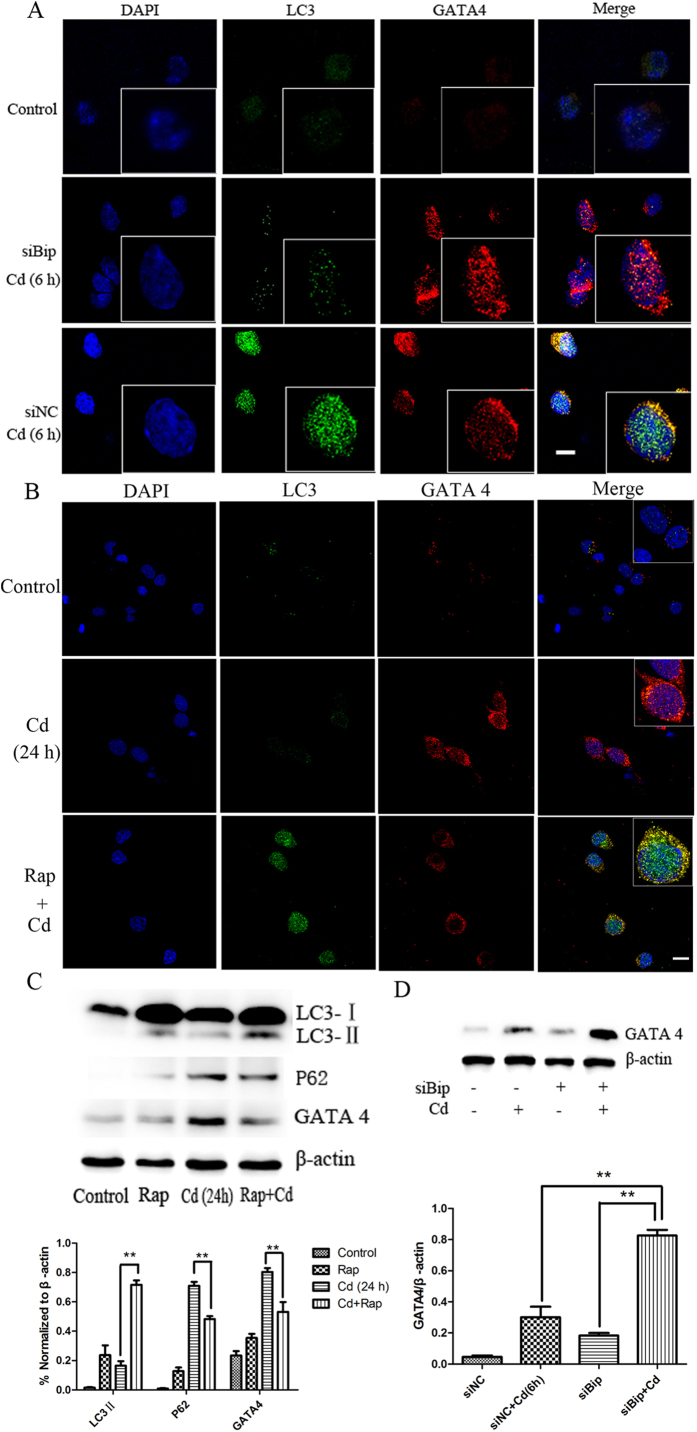
Autophagy suppresses GATA4 in Bip-dependent way. PC12 cells were transfected with either siNC or siBip and subjected to Cd treatment for 6 h (**A**) or treated with Cd in the presence or absence of rapamycin (**B**). The expression of LC3 (green) or GATA4 (red) was observed by immunofluorescence confocal microscopy. Untreated PC12 Cells were used as control. (**C**) PC12 cells were treated with Cd and/or rapamycin, and the expression of LC3, P62, and GATA4 was determined by western blot. (**D**) Effect of Cd in the presence or absence of Bip on GATA4 was evaluated by Western blotting. β-actin was used as a loading control (**p < 0.01 compared to the control group, Scale bars: 10 μm).

To test the regulation of GATA4 by selective autophagy activation, we observed the colocalization of GATA4 and LC3 in Cd–exposed PC12 cells at 6 h by immunofluorescence microscopy (Fig. 8A). GATA4 colocalised with LC3 in cells exposed to Cd for 6 h. Bip siRNA enhanced GATA4 expression and decreased the co-localization, which was confirmed by Western blot (Fig. 8C). In order to explore the association between GATA4 and autophagy, the colocalization of LC3 and GATA4 was also assessed in Cd-exposed PC12 cells with or without rapamycin at 24 h (Fig. 8B). The results indicated reduced LC3 but increased GATA4 at 24 h, suggesting a deregulated autophagy. However, the colocalization of LC3 and GATA4 was increased by pretreatment with rapamycin (Fig. 8B). Furthermore, GATA4 protein degradation via autophagy and the association with Bip was further confirmed by Western blot analysis (Fig. 8C,D). These results indicate that GATA4 appears to be targeted for autophagic degradation in Cd-exposed neuronal cells; GATA4 upregulation is possibly mediated through reduced association with Bip-regulated autophagy.

### ROS is not involved in Cd-induced autophagy and neuronal senescence

To explore whether ROS take part in Cd-induced autophagy and neuronal senescence, ROS scavenger NAC was performed. Culturing PC12 cells with 10 μM Cd in the presence of 100 μM NAC could not increase or decrease the number of positive SA-β-Gal staining cells significantly comprared with Cd group (Supplementary 1A). Similarly, there was no significant difference of the protein expression of LC3, P21 and GATA4 between the two groups (Supplementary 1B and C). Moreover, culturing primary neurons with 10 μM Cd in the presence of 100 μM NAC could not change the gene expression of IL1α or IL6 compared with Cd group (Supplementary 1D). All the results demonstrate that ROS is not involved in Cd-induced autophagy and neuronal senescence in this model.

## Discussion

Our study is the first demonstration as far as we can determine that Cd treatment induces autophagy in primary neurons and PC12 cells by enhancing ER stress and activating AMPK. Autophagy induced by Cd treatment was mediated by activation of ER stress and AMPK activation.

As neurons are terminally differentiated, one of the critical hallmarks of replicative senescence, the inability to divide, does not apply. Campisi claimed that because neurons are terminally differentiated, they cannot senesce^62^. However, in 2009, Golde and Miller postulated that neurons undergo physiological senescence, which is accelerated in AD and other neuropathies by inflammatory and oxidative stimuli^17^. Neuronal senescence induced by Cd was established in our model, with the identification of hallmarks of physiological senescence. However, cross regulation between apoptosis and cellular senescence is far from understood and further studies are needed to clarify their relationship in our model.

A recent study has shown that senescent keratinocytes die by autophagic cell death, a pathway characterized by an increase in macroautophagic activity^63^. Increasing evidence shows that autophagy is neuroprotective against various insults to the CNS^64^. Furthermore, a number of studies have demonstrated autophagy was involved in the regulation mechanism of the cell cycle arrested senescence^65^. Kang *et al*. reported that Beclin-1 and ATG7 levels were decreased in senescent cells^66^, suggesting that the initiation step of autophagy and the elongation step of the autophagosome might be damaged in senescent cells, as previously reported^67^. However, we found that the level of senescence in the siAtg5 cells showed a larger increase relative to normal cells. In addition, mice with a mosaic deletion of Atg5 develop progressive deficits in motor function from neurodegeneration^68^ and benign liver adenomas^69^. Our study is in agreement with a previous study showing that active autophagy is a potential mechanism by which p53 suppresses cellular senescence^70^.

In ER stress, autophagy is generally considered as a cytoprotective response to the overload of unfolded or misfolded proteins that exceed the capacity of proteasome^71^,^72^,^73^. There are compelling data to indicate that ER stress prevents cells from senescence by upregulating autophagy: ER stress was reported to induce cell death through stimulation of autophagy^61^, while data from rat hippocampal neurons indicate that ER stress downregulates activity of AKT and mTOR, triggering apoptosis^74^. Other evidence indicates that induction of ER stress decreased the rate of protein synthesis and triggered G1 cell cycle arrest^75^. A major discrepancy is that the extent of stress that may play a crucial role in deciding the direction of the UPR and thus the cell fate. Han *et al*. discovered that ER stress inhibited cell cycle progression via induction of p27 in melanoma cells^76^. Activation of ER stress in our model was associated with an increase in the expression of Bip, chop eIf2α and ATF-4. Liu *et al*. reported that ER stress regulated ATF4/p16 signalling on the premature senescence^77^. Kitamura and Hiramatsu reported that induction of Bip by Cd is not evident in the brain^78^. However, we identified that activation of Bip triggered by Cd was connected to autophagy and senescence in both primary neurons of rat and PC12 cells, suggesting that Cd can induce Bip-mediated autophagy in the brain. Furthermore, we found that neuronal senescence caused by Cd was attenuated as ER stress activated a protective autophagic response, further supported by other’s findings that Bip deficiency blocked LC3 conversion, and the ER is massively expanded and disorganized^79^,^80^. Therefore we conclude that the impact of stress-responsive autophagy may vary depending on cell type and how cells are exposed to the stress. Cells may activate UPR to transmit survival signals and overcome the adverse conditions when ER stress is moderate and recoverable, but when ER stress is prolonged and is too severe to be relieved by autophagy (i.e. long-time drug treatment), ER stress leads to the proapoptotic autophagy pathway. Development of new drugs that target the linkages between UPR and autophagy will have significant therapeutic benefits in the treatment of neurodegenerative diseases.

ER stress not only activates UPR, but also AMPK, which is known to regulate autophagy^81^. Our results showed that Cd treatment activated AMPK, which was inhibited by mithramycin in both primary neurons and PC12 cells^31^. Genetically silencing Bip inhibited the activation of AMPK, blocking phosphorylation at Thr172, along with the increase of p-AKT and p-p70s6k. These results, combined with the finding that suppression of Bip induced corresponding changes in the AMPK/mTOR pathway, suggest that Bip may activate autophagy through AMPK/mTOR. GATA4 was originally identified as a transcriptional regulator, which possesses a zinc-finger domain. Kang *et al*. identified that GATA4 as a key switch to regulate senescence, independently of p53 and p16^INK4a52^. We found that GATA4 plays a critical role in the neuroprotection of Bip-dependent autophagy in our model. The inhibitory effect of Bip siRNA on LC3, leading to GATA accumulation after Cd treatment implies that the preventive effect of autophagy on GATA4 is Bip-dependent. In our previous study, we found that 20 μM Cd induced ROS followed by autophagy in primary neurons at 6 h^18^. Specifically, ROS could be a upstream signal to regulate autophagy at the early stage of Cd treatment. In this study, 10 μM Cd was used to explore the effect of autophagy in neuronal senescence, in which we identified that Bip play an important role. Moreover, we found that ROS scavenger NAC could not reverse the effects autophagy, senescence, and activation of GATA4 triggered by Cd. The results indicate that Bip regulates autophagy and senescence independent of oxidative stress in neuronal cells with 10 μM Cd treatment.

In conclusion, we demonstrate that ER stress deficiency blocked autophagy flux and caused senescence in Cd-exposed neuronal cells, supporting the hypothesis that autophagy is a cellular self-defence response to Cd-induced ER stress and can subsequently alleviate cell senescence. Autophagy prevented Cd-induced cell senescence not only by inhibiting the two core pathways via Bip and AMPK, but also by suppressing GATA4. Currently, it is unknown whether any other signalling molecules are involved in Cd-induced neuronal senescence. Furthermore, whether suppression of Bip could trigger GATA4-mediated inflammation through autophagic inhibition in primary cultured cortical neurons remains to be explored. Premature senescence triggered by Cd may play a crucial role in the development of neuronal degenerative diseases. Autophagy represents an additional potential link between senescence and neurodegeneration. Our findings underscore the idea that autophagy may provide a new target for the prevention of Cd-induced neurodegenerative disorders.

## Additional Information

**How to cite this article**: Wang, T. *et al*. The ER stress regulator Bip mediates cadmium-induced autophagy and neuronal senescence. *Sci. Rep.* **6**, 38091; doi: 10.1038/srep38091 (2016).

**Publisher's note:** Springer Nature remains neutral with regard to jurisdictional claims in published maps and institutional affiliations.

## Supplementary Material

Supplementary Information
