# Supplementary material for: The ER stress regulator Bip mediates cadmium-induced autophagy and neuronal senescence
Source: Sci Rep. 2016 Dec 1;6:38091. doi: 10.1038/srep38091 (PMC5131476; doi:10.1038/srep38091)
Supplement: Supplementary Information [file srep38091-s1.pdf]

The ER stress regulator Bip mediates cadmium-induced autophagy and neuronal senescence

Tao Wang[a,b,c], Yan Yuan[a,b,c], Hui Zou[a,b,c], Jinlong Yang[a,b,c], Shiwen Zhao[a,b,c], Yonggang Ma[a,b,c], Yi Wang[a,b,c], Jianchun Bian[a,b,c], Xuezhong Liu[a,b,c], Jianhong Gu[a,b,c], Zongping Liu [a,b,c] *& Jiaqiao Zhu [a,b,c]*

[a] College of Veterinary Medicine, Yangzhou University, Yangzhou 225009, PR China;

[b] Jiangsu Co-innovation Center for Prevention and Control of Important Animal Infectious Diseases and Zoonoses,Yangzhou 225009, PR China;

[c] Jiangsu Key Laboratory of Zoonosis

* Corresponding author.

Tel.: +86 514 87991448；

Fax: +86 51487972218

E-mail: liuzongping@yzu.edu.cn，jqzhu1998@163.com

Supplementary Figure 1

PC12 Cells

A

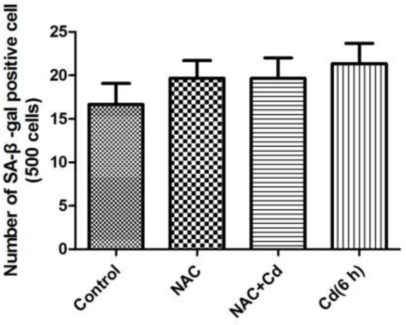

B

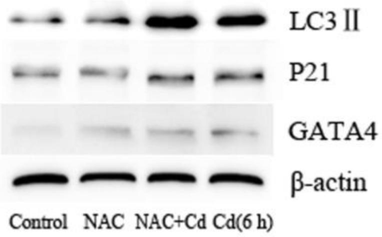

C

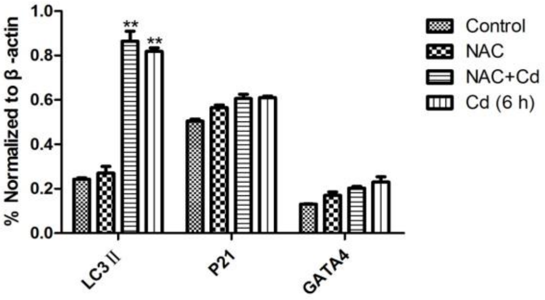

Primary neurons

D

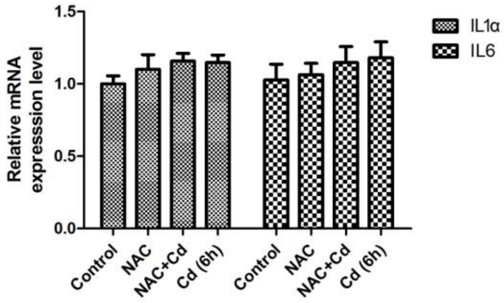

Supplementary Figure 2

Primary neurons

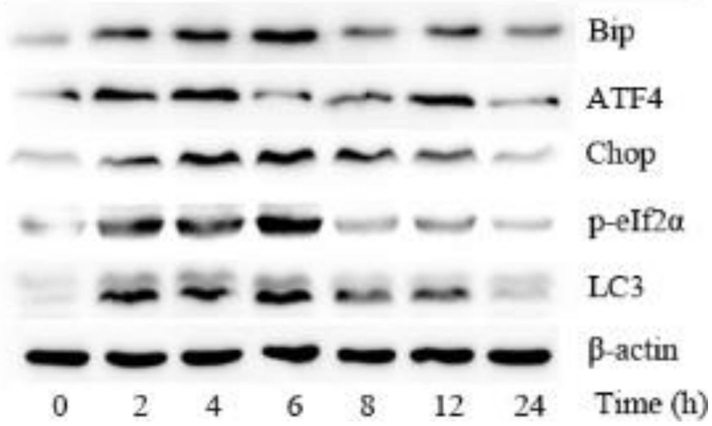

Supplementary information figure legends

Supplemental Fig. 1: ROS is not involved in Cd-induced autophagy and neuronal senescence.

(A) PC-12 cells treated with Cd for 6 h in the presence or absence of 100 μM NAC 1 h pretreatment were assayed for SA-β-Gal staining. (B) Western blot analysis for LC3, P21 and GATA 4 protein expression. The blots were probed for β-actin as a loading control. (C) Blots for LC3, P21 and GATA 4 were semi-quantified using Image Lab. (D) Primary neurons were treated as indicated in (A), and IL1α and IL6 mRNA expression were analysed by RT-PCR (** $p < 0.01$ compared to the control group).

Supplemental Fig. 2: Another Westernblot analysis of Fig. 3A.

Primary neurons were treated with 10 μM Cd for 0-24 h. Whole cell lysates were analysed by western blotting for the expression of Bip, ATF4, Chop, p-eIf2α and LC3. β-actin was used as a loading control.
